# Hidden Genetic Diversity in an Asexually Reproducing Lichen Forming Fungal Group

**DOI:** 10.1371/journal.pone.0161031

**Published:** 2016-08-11

**Authors:** Ruth Del-Prado, Pradeep Kumar Divakar, H. Thorsten Lumbsch, Ana M. Crespo

**Affiliations:** 1 Departamento de Biología Vegetal II, Facultad de Farmacia, Universidad Complutense, Madrid, Spain; 2 Science and Education, Field Museum, Chicago, Illinois, United States of America; National Cheng Kung University, TAIWAN

## Abstract

Asexual species with vegetative propagation of both symbiont partners (soredia) in lichens may harbor lower species diversity because they may indeed represent evolutionary dead ends or clones. In this study we aim to critically examine species boundaries in the sorediate lichen forming fungi *Parmotrema reticulatum–Parmotrema pseudoreticulatum* complex applying coalescent-based approaches and other recently developed DNA-based methods. To this end, we gathered 180 samples from Africa, Asia, Australasia, Europe, North and South America and generated sequences of internal transcribed spacer of nuclear ribosomal DNA (ITS) and DNA replication licensing factor MCM7 (MCM7). The dataset was analysed using different approaches such as traditional phylogeny–maximum likelihood and Bayesian–genetic distances, automatic barcode gap discovery and coalescent-based methods–PTP, GMYC, spedeSTEM and *Beast–in order to test congruence among results. Additionally, the divergence times were also estimated to elucidate diversification events. Delimitations inferred from the different analyses are comparable with only minor differences, and following a conservative approach we propose that the sampled specimens of the *P*. *reticulatum*–*P*. *pseudoreticulatum* complex belong to at least eight distinct species-level lineages. Seven are currently classified under *P*. *reticulatum* and one as *P*. *pseudoreticulatum*. In this work we discuss one of only few examples of cryptic species that have so far been found in sorediate reproducing lichen forming fungi. Additionally our estimates suggest a recent origin of the species complex–during the Miocene. Consequently, the wide distribution of several of the cryptic species has to be explained by intercontinental long-distance dispersal events.

## Introduction

Lichenized fungi form mutualistic associations either with green algae or cyanobacteria and reproduce sexually by ascospores or asexually by diaspores most commonly being isidia or soredia, which disperse both partners. Traditional criteria to delimit species are based on phenotypical characters, but in groups with limited morphological features, i.e. lichen-forming fungi, this approach has shown to underestimate species diversity in specific cases. Molecular studies have advanced our knowledge of cryptic diversity in lichens (reviewed in [1–3]). Identification of species boundaries has improved recently since ecological, biogeographical, morphological and chemical data are combined with molecular phylogenetic analyses. Analytical improvements in DNA-based approaches are playing an important role in the recognition of species diversity in lichenized fungi that otherwise would be difficult to discern using only classical phenotypic characters [3].

Previous studies have shown the presence of cryptic species in numerous species complexes especially in Parmeliaceae, which is one of the most diverse families of lichen-forming fungi [4, 5]. This includes sexually reproducing species complexes, such as *Parmelina quercina* or *Melanohalea multispora* [6–8], isidiate asexually reproducing *Melanelixia fuliginosa* or *Parmelia saxatilis* [9–11] or the *Letharia columbiana/vulpina* complex with both reproductive modes [12, 13]. However, comparatively fewer numbers of cryptic species have been found in sorediate–asexually–reproducing lichen forming fungi [14–16]. Further, sorediate species may harbor lower species diversity because they may indeed represent evolutionary dead ends or clones [17–20].

Previous studies suggested the presence of cryptic lineages in the sorediate *Parmotrema reticulatum*–*Parmotrema pseudoreticulatum* complex but the sampling was insufficient for conclusive results [15, 21]. *Parmotrema reticulatum* was described in 1836 from Ireland but the name has subsequently been used for foliose lichens with a minutely reticulate-maculate and sorediate upper surface, black lower surface, simple to branched rhizines containing atranorin and salazinic acid from a wide range of tropical and subtropical habitats [22, 23]. The species has a wide ecological amplitude, occurs most frequently as an epiphyte, more rarely on siliceous rocks, and normally reproduces asexually by non-corticated, symbiotic dispersal units, called soredia. *Parmotrema pseudoreticulatum* was separated later for populations in coastal areas of the south of the Iberian Peninsula, Balearic and Canary Islands and Morocco that differ from *P*. *reticulatum* by subtle morphological characters [21, 24]. It grows mainly on *Quercus* and *Olea* tree trunks. The species was considered synonymous with *P*. *reticulatum* by several authors [25, 26] but was accepted by others [27, 28].

Molecular data supported the separation of the two species [15] and moreover suggested that additional cryptic lineages exist within this complex. More recently, Del-Prado and colleagues [21] using ITS data explored the genetic diversity in *P*. *reticulatum* and *P*. *pseudoreticulatum* and supported previous findings that these species include several distinct lineages.

Coalescent based approaches have shown to be well suited to critically evaluate species diversity in fungi [3]. Further, these methods can accurately display relationships when incomplete lineage sorting and gene tree heterogeneity hide phylogenetic relationships among species [29, 30]. Commonly used methods to critically evaluate species delimitation include poisson tree processes (PTP) model [31], the automatic barcode gap discovery (ABGD) [32], and the general mixed Yule coalescent model (GMYC) [33, 34], and SpedeSTEM [35].

PTP infers putative species boundaries on a given non-ultrametric phylogenetic input tree. Speciation rate is modelled by directly using the number of substitutions, assuming that the number of substitutions between species is higher than those within species. This model is mainly intended for delimiting species in single-locus phylogenies.

ABGD provides an efficient algorithm that allows partitioning a DNA sequence dataset into clusters of like taxa, i.e. candidate species or ‘primary species hypotheses’, according to a range of potential barcode gap thresholds. In ABGD analyses, potential threshold values are obtained from the data themselves (not *a priori*), and different clades within the same dataset may be assigned different thresholds.

The GMYC model allows locating of nodes that define the transitions between intraspecific and interspecific relationships on a chronogram. This method uses branch length differences to identify nodes that circumscribe species. This model assumes that the independent evolution leads to appearance of genetically distinct clusters, which are separated by long internal branches. A number of studies have employed this coalescent approach to successfully delineate species and detect cryptic species in various groups of organisms [33, 36–38].

SpedeSTEM uses maximum likelihood and information theory to evaluate phylogenetic models of lineage composition, enables biologists to identify distinct evolutionary lineages shortly after their formation. This approach requires operational taxonomic units (OTUs) to be designated a priori.

Additionally, putative species were evaluated based on the threshold of 0·016 substitutions per site (s/s) that separate intra- and interspecific distances in parmelioid lichens [39].

Moreover, estimates of divergence dates of species is a major step to elucidate potential ecological, biogeographic and climatic patterns that drive diversification and explain the current distribution of species. In lichen-forming fungi, limited data are available on species turnover, diversification events and subsequently on factors driving diversification [8, 9, 40–44]. This is mainly due to the poor fossil record for lichen-forming fungi and due to uncertainties in the interpretation of the few known fossil records [45–47]. It has been demonstrated that dating speciation events using priors on substitutions rates in a full probabilistic coalescent species tree framework–the approach implemented in *Beast—is appropriate for studies on taxonomic groups with poor fossil records [48, 49].

We here aim to critically examine species diversity in the locally frequent *P*. *reticulatum–P*. *pseudoreticulatum* complex. Specifically, we aim to: i) delimit species applying coalescent-based species delimitation methods, and ii) elucidate divergence times. To this end, we gathered 180 samples from Africa, Asia, Australasia, Europe, North and South America and generated sequences of internal transcribed spacer of nuclear ribosomal DNA (ITS) and DNA replication licensing factor MCM7 (MCM7).

## Material and Methods

### Taxon sampling

Sequence data of the ITS and the protein-coding MCM7 genes were analyzed in 28/21 specimens of *P*. *pseudoreticulatum* and 152/121 of *P*. *reticulatum*, collected from distant geographic regions throughout the species distributions. Specimens were collected in locations outside of national parks or protected areas therefore no specific permissions were required. Also this study does not involve endangered or protected species.

*Parmotrema cetratum* was selected as outgroup since it has been shown to be closely related [50]. For ITS, data from 94 individuals were generated for this study and 88 sequences downloaded from GenBank. For MCM7, data from 144 sequences was generated for this study. Detailed collection information and GenBank accession numbers are listed in S1 Table.

### DNA extraction, PCR and sequencing

Total DNA was extracted from freshly collected materials and frozen specimens, using the DNeasy Plant Mini Kit (Qiagen) following the instructions of the manufacturer, with the slight modifications described previously [51].

Genomic DNA (5–25 ng) was used for PCR amplifications of the ITS and MCM7 regions. Standard PCR amplifications were conducted in 25-μL reaction volumes. Primers, PCR and cycle sequencing conditions for nuclear ITS were the same as described previously [21].

Primers X-Mcm7-F and Mcm7-1348rev [52, 53] were used to amplify the MCM7 marker. PCR amplifications were carried out in an automatic thermocycler (Techne Progene, Jepson Bolton & Co. Ltd., Waltford, Herts, UK) using the following conditions: initial denaturation at 94°C for 10 min followed by 40 cycles at 94°C for 45 sec, 56°C for 1 min, and 72°C for 1 min. A final extension step at 72°C for 8 min was added, after which the samples were kept at 4°C. Amplification products were visualized on 1% agarose gels stained with SYBR^®^ Safe DNA (Life Technologies Corporations, USA) gel stain (10 000× concentrated in DMSO) and subsequently purified using the enzyme exoSAP-IT (GE Healthcare, UK) according to the manufacturer’s instructions. Both complementary strands were sequenced using Big Dye Terminator reaction kit (ABI PRISM, Applied Biosystems). Cycle sequencing reactions were performed with the same sets of primers used in the amplification step. Sequencing reactions were electrophoresed on a 3730 DNA analyzer (Applied Biosystems) at the Unidad de Genómica (Parque Científico de Madrid).

### Sequence alignment

Sequence fragments generated for this study were assembled and edited using the program SeqMan v.7 (Lasergene R, DNASTAR, Madison, Wisconsin, USA). Sequence identity was confirmed using the mega-BLAST search function in GenBank. We used the program MAFFT v.6 [54] with the parameters set to default values to align the DNA sequences for each data set separately. Ambiguously aligned positions were identified and removed using G-Blocks implementing the options for a less stringent selection [55].

### Phylogenetic analyses

The separate alignments and the combined data set were analyzed using maximum likelihood (ML) and a Bayesian Markov chain Monte Carlo (B/MCMC) approach.

We examined nodes to identify well-supported (ML bootstrap values > 70%) conflicts among the individual ITS and MCM7 phylogenies before combining the alignments [56, 57].

The ML analysis was performed using an online version of the program RAxML v.7.0.4 (http://phylobench.vital-it.ch/raxml-bb/) [58, 59], assuming a GTRGAMMA model which includes a parameter (Γ) for rate heterogeneity among sites and chose not to include a parameter for estimating the proportion of invariable sites. Nodal support was evaluated using 1000 bootstrap pseudoreplicates. For the combined data set we use a locus-specific model partitions in RAxML. Both loci were treated as separate partitions.

Separate and concatenated data sets were also analyzed with Bayesian inference as implemented in MrBayes v3.1.2 [60]. Models of DNA sequence evolution for each locus were selected with the program jModeltest v0.1 [61], using the Akaike information criterion (AIC) [62]. The model TIM2ef+G was selected for ITS; and SYM+I for the MCM7 region. The combined data set was partitioned into the two parts (ITS, MCM7), and each partition was allowed to have its own parameters [63]. No molecular clock was assumed. Two parallel runs of 3 million generations were done, starting with a random tree and employing 12 simultaneous chains each. Every 1000^**th**^ tree was saved into a file. The first 300,000 generations (i.e., 3000 trees) were deleted as the “burn-in” of the chains. The outputs of MrBayes were examined with the program Tracer v1.5 [64] to check for convergence of different parameters, determine the approximate number of generation at which log likelihood values stabilized and identify the effective sample size (ESS) for each parameter. Topological convergence in the two independent MCMC runs was checked with the “compare” plots in the program AWTY [65]. Posterior probabilities (PPs) of clades were obtained from the 50% majority-rule consensus of sampled trees after excluding the initial 10% as burn-in. Only clades that received bootstrap support equal to or above 70% in ML analyses and posterior probabilities equal to or above 0.95 were considered as strongly supported.

Phylogenetic trees were drawn using the program TREEVIEW v.1.6.6 [66].

Alignments are available at TreeBase (http://www.treebase.org) under study accession number S19485, and phylogenetic trees under accession numbers 38072, 38073 and 38074.

### Calculation of genetic distances

Pairwise ML distances (given as the number of nucleotide substitutions per site) among the ITS rDNA sequences of the *Parmotrema reticulatum*–*P*. *pseudoreticulatum* complex were calculated (S1 Table). Genetic distances were calculated with TREE-PUZZLE 5.2 [67] using the GTR model of nucleotide substitution, assuming a discrete gamma distribution with six rate categories. The program generates an output file which consists of a triangular matrix with all pairwise distances between all the samples included. This matrix was visualized with the Microsoft Office program Excel 2000 and genetic distances between different specimens of the *P*. *reticulatum*–*P*. *pseudoreticulatum* complex were manually identified. Candidate species were proposed based on the threshold of 0·016 substitutions per site (s/s) that separate intra- and interspecific distances in parmelioid lichens [39]. The distance values in the matrix ≤ 0·016 s/s have been considered the values between the samples of the single species. The filter provided by Microsoft Excel was applied to separate values ≤ 0·016, obtaining for every specimen included in the analysis the group of specimens with which it shares the values that characterize the species range.

### Automatic Barcode Gap Discovery (ABGD) analyses

This is an automatic procedure that sorts the sequences into hypothetical species based on the barcode gap. This method automatically finds the distance where the barcode gap is located [32].

The ABGD method was carried out for the ITS dataset using the Web interface at http://wwwabi.snv.jussieu.fr/public/abgd/abgdweb.html. Default parameters were chosen using Kimura 2-parameter (K2P) distances that correct for transition rate bias (relative to transversions) in the substitution process. The default for the minimum relative gap width was set to different values between 0.1 and 0.15.

### Poisson tree processes (PTP)

PTP does not require an ultrametric tree, as the transition point between intra- and inter-specific branching rates is identified using directly the number of nucleotide substitution [31]. PTP incorporates the number of substitutions in the model of speciation and assumes that the probability that a substitution gives rise to a speciation event follows a Poisson distribution. The branch lengths of the input tree are supposed to be generated by two independent classes of Poisson events, one corresponding to speciation and the other to coalescence. The ML phylogeny obtained with RAxML of the ITS data set, was used as the input trees to run PTP species delimitation analysis in the PTP webserver (http://species.h-its.org/ptp/). We ran the PTP analysis for 100,000 MCMC generations, with a thinning value of 100 a burn-in of 25%. Outgroup taxa were removed for species delimitation.

### General Mixed Yule Coalescent (GMYC) species delimitation

This method requires a fully resolved tree with branch lengths estimates. It is based on the differences in branching rates between interspecific branching events and intraspecific relationships in a chronogram.

For the analyses, the ML tree obtained from a RAxML search using the ITS was used to infer an ultrametric tree using the program BEAST v.1.8.0 [68]. For this analysis identical haplotypes were removed. We used a site-specific GTR substitution matrix and a gamma distributed model of among-site rate heterogeneity with four discrete rate categories. We implemented an uncorrelated relaxed lognormal clock [69], and selected a Yule tree prior. Default values were used for remaining priors. MCMC analysis was run for a total of 10 million generations, sampling every 1000steps and excluding the first 10 million generations of each run as burn-in. Convergence was assessed by examining the likelihood plots through time using TRACER v1.4 [64].

GMYC requires a fully dichotomous chronogram and thus we used multdivtime to convert our chronogram into a fully dichotomous chronogram with internal branches of length zero, where appropriate. The ITS chronogram was then analyzed using the GMYC package in SPLITS in R (version 2.10, www.cran.r-project.org), using single and multiple threshold approaches [33, 34].

The two outgroup samples (*P*. *cetratum*) were excluded from the data set using the drop.tip command in ape [70].

After optimization, we plotted the lineage through time (LTT) plot [71] with the threshold indicated and a chronogram that had the putative species indicated. Finally, we used the summary command to summarize the output statistics, including the results of the Likelihood ratio test (LRT) and the indication of the numbers of clusters and entities.

### Validation approach: SPEDEStem

We used spedeSTEM v1.0 [35] as a validation approach to evaluate support for the candidate species previously delimited by PTP, ABGD, genetic distances and GMYC. This method calculates the probability of different models of lineage composition using maximum likelihood and evaluates these models using information theory [72].

SpedeSTEM takes as input ultrametric gene trees from independent loci that were inferred from the ML trees obtained from a RAxML search and using the program BEAST, as described above. SpedeSTEM requires an estimate of θ in order to scale the branch lengths in the species trees it produces. Using DnaSPv5 [73] we computed the average θ across the two included loci (theta = 0.03), with a scaling (segregating sites/total sites) of 1:1.6 for ITS and MCM7 respectively.

Analyses were run online in the SpedeSTEM server at: https://spedestem.osu.edu/runspedestem. A table of models ranked by model probability is returned.

A total of seventeen species delimitation scenarios were tested and validated through spedeSTEM. These candidate species were related with those proposed in our previous study [21]. Twelve of these putative species were selected from those identified in the GMYC_multiple_ approach from the combined data set, as the less conservative scenario (data not shown). The remaining five were selected from the supported groups nested in the clade A2 in the phylogenetic concatenated tree.

### Species tree and Divergence time estimates

We used the coalescent-based hierarchical Bayesian model *BEAST implemented in BEAST 1.8.0, as described elsewhere [49], to estimate a species tree following our proposed species delimitation scenario and to infer divergence dates using a coalescent-based species tree approach. *BEAST estimates the species tree directly from the sequence data, and incorporates the coalescent process, uncertainty associated with gene trees, and nucleotide substitution model parameters [74]. Further, species tree methods incorporating the process of gene lineage coalescence likely provide a more biologically realistic framework for dating divergence events [48]. In the absence of relevant fossil evidence for the *P*. *reticulatum–P*. *pseudoreticulatum* complex, we used the molecular evolution rates for the ITS marker [2.43 x 10−^9^ substitution/site/year (s/s/y)] recently reported for the related lichen-forming genus *Melanelixia* [9] to estimate the time to the most recent common ancestor (MRCA) for the clades. Implementing an uncorrelated relaxed lognormal clock, we selected a Yule process and gamma-distributed population sizes for the species-tree prior and a piecewise linear population size model with a constant root. Default values were used for remaining priors.

Two independent Markov chain Monte Carlo (MCMC) analyses were run for a total of 50 million generations, sampling every 1000 steps and excluding the 12500 trees as burn-in. We assessed convergence by examining the likelihood plots through time using Tracer and compared summarized tree topologies from separate runs; the effective sample sizes (ESS) of parameters of interest were all above 200. The posterior probabilities of nodes were computed from the sampled trees (excluding burn-in samples) using TreeAnnotator 1.8.0 [68].

While most of the putative species were represented by multiple individuals, the other three had two to three individuals. In such cases, species delimitation approach could be biased and caution must be taken. “Bayesian Phylogenetics and Phylogeography” (BP&P) [75], implementing the nearest neighbor interchange algorithm, could be a reliable method to handle disproportionate sampling, however, this approach requires a multi-locus dataset. Since we had only two loci in our dataset, we refrained from using this approach.

## Results

### Phylogenetic analyses

The ITS data matrix included 180 OTUs of the *P*. *reticulatum*–*P*. *pseudoreticulatum* complex and 462 unambiguously aligned nucleotide positions (Tree- BASE No. S19485). 94 sequences were newly generated (S1 Table). For the Bayesian analysis the LnL value was -1851.772 with a standard deviation of ±318.04, and for ML the LnL value was -1553.1812.

The MCM7 data matrix included 142 OTUs of the *P*. *reticulatum*–*P*. *pseudoreticulatum* complex and 610 unambiguously aligned nucleotide positions (Tree- BASE No. S19485). 144 sequences were newly generated (S1 Table). For the Bayesian analysis the LnL value was -1885.326 with a standard deviation of ±316.98, and for ML the LnL value was -1581.497.

The partitioned ML analysis of the concatenated data matrix yielded the optimal tree with Ln likelihood value = -3321.011. The mean LnL value of the two parallel run of the Bayesian analysis for the two combined loci was -3682.9 with a standard deviation of ±0.55.

**Fig 1:**
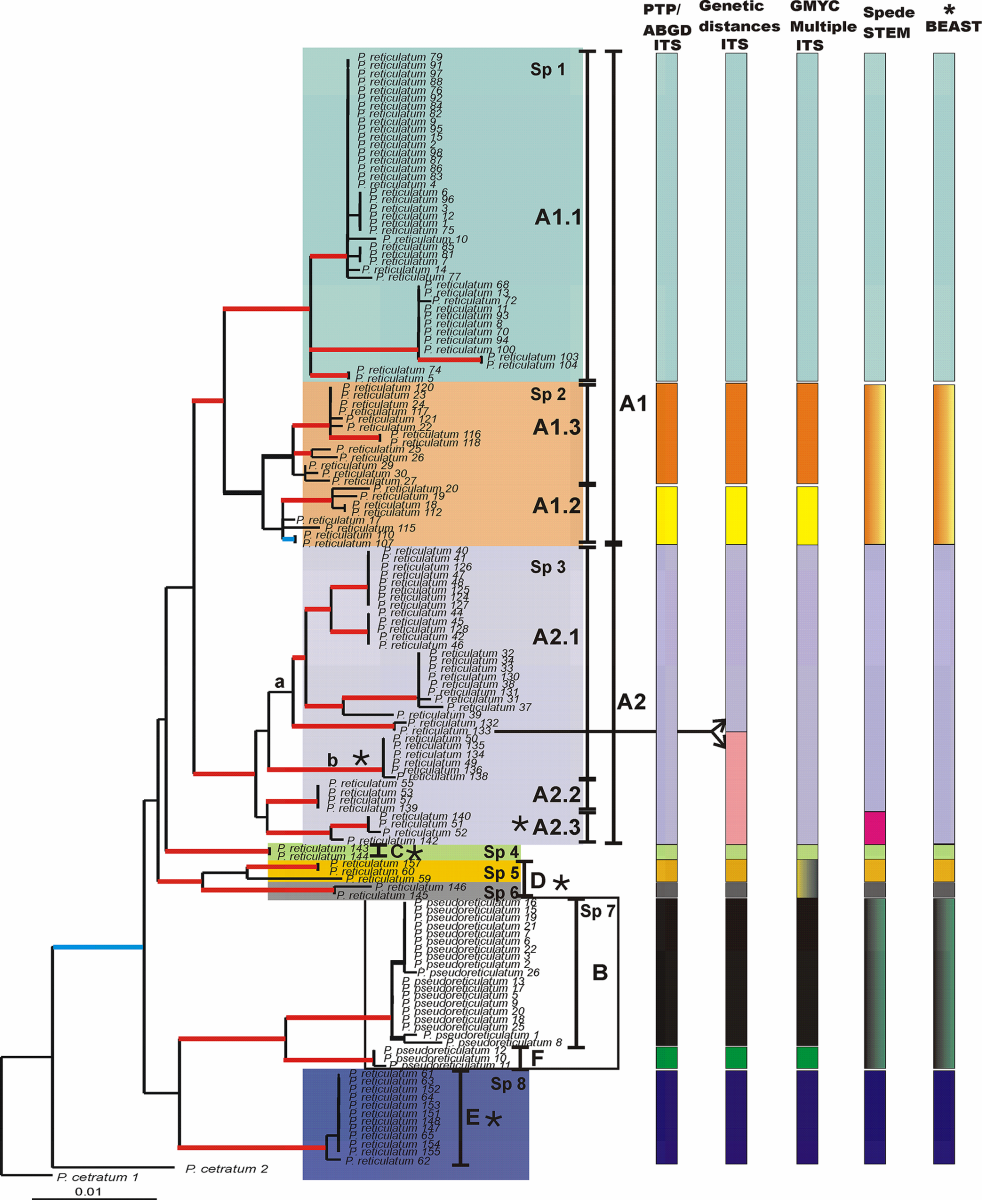
Phylogenetic relationships in the *Parmotrema reticulatum*–*Parmotrema pseudoreticulatum* complex. Maximum-likelihood phylogenetic tree inferred from two loci (ITS and MCM7). Branches that received strong support (bootstrap values ≥ 70%, posterior probabilities ≥ 0.95) in any of two analyses RaxML, and B/MCMC are in boldface. The branch that received strong support only in the ML analysis is indicated by a blue boldface line, whereas branches that received strong support only in the B/MCMC analysis are indicated by a black boldface line. Branches that were strongly supported in both analyses are indicated by a red boldface line. Species delimitation scenarios obtained from different methods are indicated in columns to the right (details discussed in text). The 8-species scenario recovered in species tree estimation using *BEAST is also indicated in the right column. The proposed 8 candidate species are highlighted with different color shade and with letters ‘Sp1’ to ‘Sp8’. Asterisks indicate clades that show gene genealogical concordance.

Since the topologies of the trees estimated from ML and Bayesian methods did not present any well-supported conflict, only ML topologies are shown with bootstrap and posterior probability values indicated on these topologies (Fig 1, S1 Fig and S2 Fig).

The topologies of the trees show that the samples of *P*. *reticulatum* and *P*. *pseudoreticulatum* split into multiple statistically supported clades. Statistical support for the different clades recovered in the concatenated analyses and in the independent ITS and MCM7 topologies are summarized in S2 Table.

In the concatenated topology, specimens of *P*. *reticulatum* were spread between two main monophyletic groups (clades A1 and A2) with strong support in both analyses. These two clades split in different monophyletic groups (Fig 1).

It is remarkable to note that clade A1.1 splits in three different supported clusters in MCM7 topology, without showing any conflict with the ITS and concatenated topologies. Samples from the clades A1.2 and A1.3, in the concatenated topology, nested in a monophyletic group supported in the Bayesian analysis. In the MCM7 topology, samples from both clades are distributed on intermixed mode in two independent supported clades.

In clade A2.2 from the MCM7 topology are nested some samples that, in ITS, are grouped with samples of clade A2.1a. Additionally the two samples from clade A2.1a that are independent in ITS, are nested in a monophyletic supported clade with the samples from clade A2.3 in MCM7.

These conflicts did not influence the compatibility of the concatenated data set.

In the three topologies, two samples from Australia and two from Chile formed a different well-supported clade (clade D). Sixteen samples from the Canary Islands clustered in two independent supported groups (clades C and E).

Clade A2 further split in two supported clades, A2.1b and A2.3, which are present in both single locus genealogies. Clades C, D and E are also present in both single-locus genealogies.

Specimens of *P*. *pseudoreticulatum* split into two well-supported monophyletic groups (clades B and F) in ITS and in the concatenated tree. Additionally in the combined topology, clades B and F are grouped in a supported clade. In MCM7 topology, all the samples of *P*. *pseudoreticulatum* are nested in a single supported monophyletic group, in which samples from clades B and F are intermixed.

Phylogenetic relationships among the clades were not resolved with statistical support.

### Identifying candidate species

The RAxML tree obtained from the concatenated data set was used to illustrate the delimitation of putative species recognized by the different approaches conducted with the ITS and with the concatenated dataset (Fig 1).

ABGD and PTP analyses applied to ITS dataset detected 10 candidate species, which correspond to the well supported clades A1.1, A1.2 A1.3, A2, B, C, E and F obtained in phylogenetic analyses. However, clade D splits in two different candidate species: the first includes samples from Chile, and the second from Australia. The GMYC approach employing the multiple threshold (GMYC_multiple_) on ITS dataset suggests almost the same candidate species as ABGD and PTP analyses with the only difference that all samples nested in clade D are included in a single species.

Genetic distance analyses in which a barcode gap is applied (0.016 s/s), also suggested the same clusters as those obtained in ABGD and PTP analyses, with one exception. Clade A2 is split into two different putative species, one of them includes the samples from the well-supported clade A2.1a, and the other grouped the well-supported clades A2.1b, A2.2 and A2.3 (Fig 1). Sample number 133 from clade A2.1a is included in both candidate species.

In both versions of the GMYC method (single and multiple threshold), the likelihood of the GMYC model was significantly higher than the likelihood of the null model. However the likelihood values of the single and multiple threshold analyses did not differ significantly. Therefore we show the results from multiple threshold analyses, which represent a more comparative approach.

All the samples nested in clade E were collected in different localities from the Canary Islands.

Clades marked with an asterisk are present in both single locus analyses.

### Validation approach: SPEDE Stem and *BEAST analyses

SpedeStem analysis proposed a 9-species delimitation scenario that corresponds to the well supported clades: A1.1, A1.2+A1.3, A2.1+A2.2, A2.3, C, B+F and E. Clade D splits into two different species, one groups samples from Chile and the other from Australia.

Finally, a species tree was estimated with *BEAST following our proposed species delimitation scenario by SPEDEStem. An 8-species scenario was recovered in species tree estimation, which corresponds to the species validated by SpedeSTEM with the exception of samples from clade A2.3 that are not validated as a separate species but they are included in the clade A2 species.

### Molecular dating analysis

**Fig 2:**
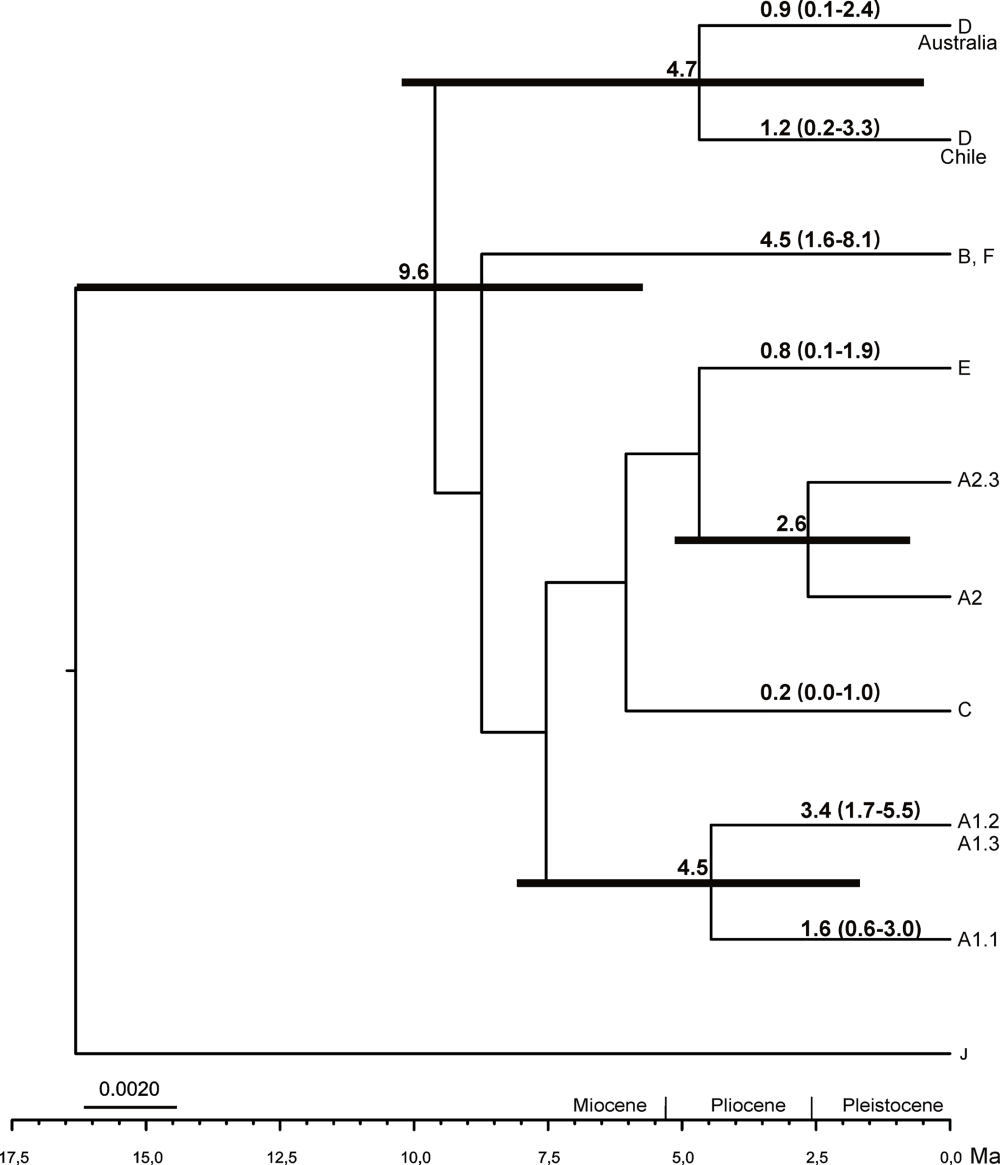
*BEAST species time tree of *P*. *reticulatum*–*P*. *pseudoreticulatum* complex diversification. The chronogram was estimated from a two-locus, (ITS and MCM7), coalescent-based species tree in *BEAST. Mean ages in millions of years (Ma), and their 95% highest posterior density (HPD) bars are shown above nodes. Clades A-E represents putative species recovered in *BEAST analysis. J = out group.

The estimated timing of diversification events in the *P*. *reticulatum*-*P*. *pseudoreticulatum* complex is shown in the rate calibrated species tree inferred using *BEAST (Fig 2). The mean node ages and divergence date ranges (95% highest posterior density intervals, HPD) of the clades are shown in the figure.

The crown of the complex was estimated at 9.6 Ma (95% HPD = 5.8–16.3 Ma) during the Miocene. Our molecular dating analyses supported three separate major divergence events that led to the origin of clades A1, A2 and D. Samples from Australia in clade D separated from those of Chile around 4.7 Ma. Clade A1.1 splits from clades A1.2 and A1.3 around 4.5 Ma. Samples from clade A2.3 diverged from the rest of clade A2 about 2.6 Ma.

The diversification of *P*. *pseudoreticulatum* (clades B and F) was estimated to be around 4.5 Ma. In a second radiation event clades A1.2 and A1.3 radiated around 3.4 Ma. Later, within clade D, samples from Chile radiated around 1.15 Ma, and those from Australia around 0.9 Ma. Clade A1.1 was estimated to have radiated by the Pleistocene (1.6 Ma). Clade E radiated around 0.8 Ma and clade C 0.2 Ma.

## Discussion

Previous molecular phylogenetic studies have provided evidence for the existence of several cryptic lineages hidden under the names of *P*. *reticulatum* and *P*. *pseudoreticulatum* [15, 21]. The specific aim of this paper was to evaluate eight cryptic lineages in *P*. *reticulatum* and two in *P*. *pseudoreticulatum* that were identified and proposed as candidate species in our previous work [21]. From an extended taxon sampling and a two locus data set, we have applied recently developed approaches used to delimitate and validate species boundaries, and in addition, we studied the timing of diversification events in the group.

In the current study six major well-supported clades were found in the combined analyses of *P*. *reticulatum*-*P*. *pseudoreticulatum* complex. These clades correspond to those previously discussed, with the exception of clade C. Additionally, in our previous studies samples of *P*. *pseudoreticulatum* fell in two independent well supported clades (B and F), whereas in the current study they formed one statistically supported monophyletic clade. Relationships among clades remained largely unsupported as in our previous studies that employed ITS data only [15, 21].

Phylogenetic analyses alone are insufficient to draw conclusions on species boundaries in this complex and additional studies have been applied. To achieve confidence in species delimitation we used an integrative taxonomic approach that uses complementary sources of data as ecology, chemistry, morphology, biogeography, and multiple molecular analytical approaches. However, in the *P*. *reticulatum*–*P*. *pseudoreticulatum* complex these data are limited as in other cryptic lineages of lichenized-fungi. Only in two clades (C and E) a geographical pattern has been found with all samples coming from the Canary Islands. For the other lineages we could not observe any correlation with geographical, morphological, chemical or ecological patterns. An increasing number of studies have revealed the presence of cryptic species in lichen-forming fungi without any recognizable phenotypical or biogeographical characterization of these clades [2, 3]. In these cases, coalescent-based approaches and other recently developed DNA-based methods have been widely used as an objective measure to delimitate and validate species boundaries [38, 76–78].

Delimitations inferred from the different analyses are comparable with only minor differences. The analyses supported between 9 and 11 candidate species in the *P*. *reticulatum*–*P*. *pseudoreticulatum* complex. Differences among methodological approaches are common [76] due to different statistical power to detect independent lineages and could indicate that assumptions of one or more of the methods are violated. In this work, differences in the circumscriptions of clades A2 and D were detected.

In clade A2 there is a conflict between PTP / ABGD and our manually calculated genetic distances. Also, the lineage labelled as “b” and group A2.3 showed gene genealogical concordance, which is taken as evidence that these clades represent reproductively isolated lineages [2, 3]. Therefore it seems that the speciation process has not been completed within this clade. When number of nucleotide substitutions are considered to assign DNA sequences to species relied on the existence of a barcode gap [76], one might expect to find overlap between inter and intra-specific divergences for clades that are speciating [76]. Genetic distance calculations proposed two species instead of the one proposed by PTP and ABGD, due to a higher statistical power to detect species lineages as we consider a specific barcode gap calculated for parmelioid lichens [31, 79].

Another difference was found in clade D. In this case, GMYC regarded it as one species, whereas other methods identified two different candidate species. In our study GMYC suggested more conservative species delimitations, which differs from other studies in which over-splitting in comparison to ABGD or PTP was found [31, 79].

Based on the proposed candidate species by the different methods tested in this work and in the proposed candidate species in our previous work, we have implemented a validation coalescent-based approach through spedeSTEM analysis, which pointed 9 species scenario as the most likely.

Finally, an 8-species scenario was recovered in species tree estimation by*BEAST. This largely corresponds to the species validated by SpedeSTEM with the exception of samples from clade A2.3 that are not validated as a separate species however they are included in the clade A2 species.

*BEAST is as a coalescent-based species tree method that can accurately depict relationships even in cases where incomplete lineage sorting and gene tree heterogeneity obscures phylogenetic relationships among species [29, 30]. However spedeSTEM may be less accurate in cases of recent speciation events [76].

With the data at hand we are following a conservative approach advocated by Carsten and coworkers [76] who argued that it would be better to fail to delimit species to falsely delimit entities that do not represent independent lineages.

We propose that the sampled specimens of the *P*. *reticulatum*–*P*. *pseudoreticulatum* complex belong to at least eight distinct species- level lineages. Seven currently classified as *P*. *reticulatum* and one as *P*. *pseudoreticulatum*. Although most analyses proposed the presence of two species lineages within *P*. *pseudoreticulatum*, we tentatively follow a conservative approach as suggested by spedeSTEM and *BEAST. The formal description of the new species will be done in a companion paper.

The *Parmotrema reticulatum* complex has traditionally been considered a purely asexually reproducing fungal group. Further, asexually reproducing species in lichens–sorediate–and in filamentous fungi in general has largely been observed as an evolutionary dead end or clones [80]. By contrast, we here demonstrate high cryptic species diversity within the *P*. *reticulatum* complex (Fig 1). Moreover, recently, there has been a growing body of evidence in many other fungal groups with a supposedly asexual lifestyle that they indeed have the ability to endure sexual reproduction; suggesting cryptic sexuality [81, 82]. Numerous criteria, such as observation of initial morphological stages of sexual reproductive organs, finding of recombination and identification of mating-type genes involved in sexual reproduction in the genome have been used to provide evidence of cryptic sexuality in putative asexual species [83, 84]. In the *P*. *reticulatum* complex, ascomata or ascomatal primordia are rarely found [22, 23]. However, given the evidence of cryptic sexuality in ascomycetes we hypothesize that cryptic sexuality may contribute to the genetic diversity and speciation in the *P*. *reticulatum* complex. A test of our hypothesis can be the search for mating-type genes responsible for sexual reproduction in the genome of that species complex.

Our divergence time estimates suggest the origin of the species complex in the Miocene (Tortonian) around 9.6 Ma. This is consistent with the estimated age of the genus *Parmotrema* [40] at around 13 Ma. Our data suggest that the major diversification events within the complex occurred during the Pliocene with more recent radiations at the tips during the Pleistocene. Although rate-calibrated divergence estimates must be interpreted with caution, these results are comparable with those found in *Melanohalea* [8]. Miocene and Pliocene were characterized by cooling and a series of climate fluctuations including ice ages and uplifting of major mountain systems in Eurasia [76]. These events accelerated turnover of the terrestrial biota and several studies have shown that the Miocene has been an important time for diversification of species groups in Parmeliaceae [76]. Specifically the origin of the genus and the species complex corresponds to an event of increasing aridity in the Miocene (15–8 Ma). This climatic event, together with habitat changes due to previously indicated mountain uplift and associated shifts in vegetation patterns could have played a key role in the origin of the genus and the diversification of the complex. Radiations within the complex occurred from the end of the Pliocene when the climate became cooler, drier and seasonal.

The cryptic species identified in our study are currently widely distributed in different areas from the Northern and Southern Hemisphere, including Europe, North Africa and Canary Islands, Australia, South America and Asia. This wide distribution has to be explained by intercontinental long-distance dispersal events given the recent origin of the species complex.

## Supporting Information

S1 Fig ITS ML phylogenetic tree. ML phylogenetic tree of *Parmotrema reticulatum*-*Parmotrema pseudoreticulatum* complex from ITS sequences. Branches that received strong support (bootstrap values ≥ 70%, posterior probabilities ≥ 0.95) in any of two analyses RaxML, and B/MCMC are in boldface. The branch that received strong support only in the ML bootstrap analysis is indicated by a blue boldface line, whereas branches that received strong support only in the B/MCMC analysis are indicated by a black boldface line. Branches that were strongly supported in both analyses are indicated by a red boldface line.
(PDF)

S2 Fig MCM7 ML phylogenetic tree. ML phylogenetic tree of *Parmotrema reticulatum*-*Parmotrema pseudoreticulatum* complex from MCM7 sequences. Branches that received strong support (bootstrap values ≥ 70%, posterior probabilities ≥ 0.95) in any of two analyses RaxML, and B/MCMC are in boldface. The branches that received strong support only in the ML bootstrap analysis are indicated by a blue boldface line, whereas branches that were strongly supported in both analyses are indicated by a red boldface line.
(PDF)

S1 Table Collection information for all the specimens included in the present study. Gen-Bank accession numbers for the two sampled loci: nuclear ribosomal internal transcribed spacer region (ITS), and DNA replication licensing factor MCM7 (mcm7). Newly generated sequences for this study are indicated in boldface.
(DOC)

S2 Table Statistical support for the clades recovered in the concatenated, ITS and mcm7 topologies. Clades do not recovered in the topology are indicated with an asterisk (*).
(DOC)
