# Supplementary figures and images for: Hidden Genetic Diversity in an Asexually Reproducing Lichen Forming Fungal Group
Source: PLoS One. 2016 Aug 11;11(8):e0161031. doi: 10.1371/journal.pone.0161031 (PMC4981466; doi:10.1371/journal.pone.0161031)

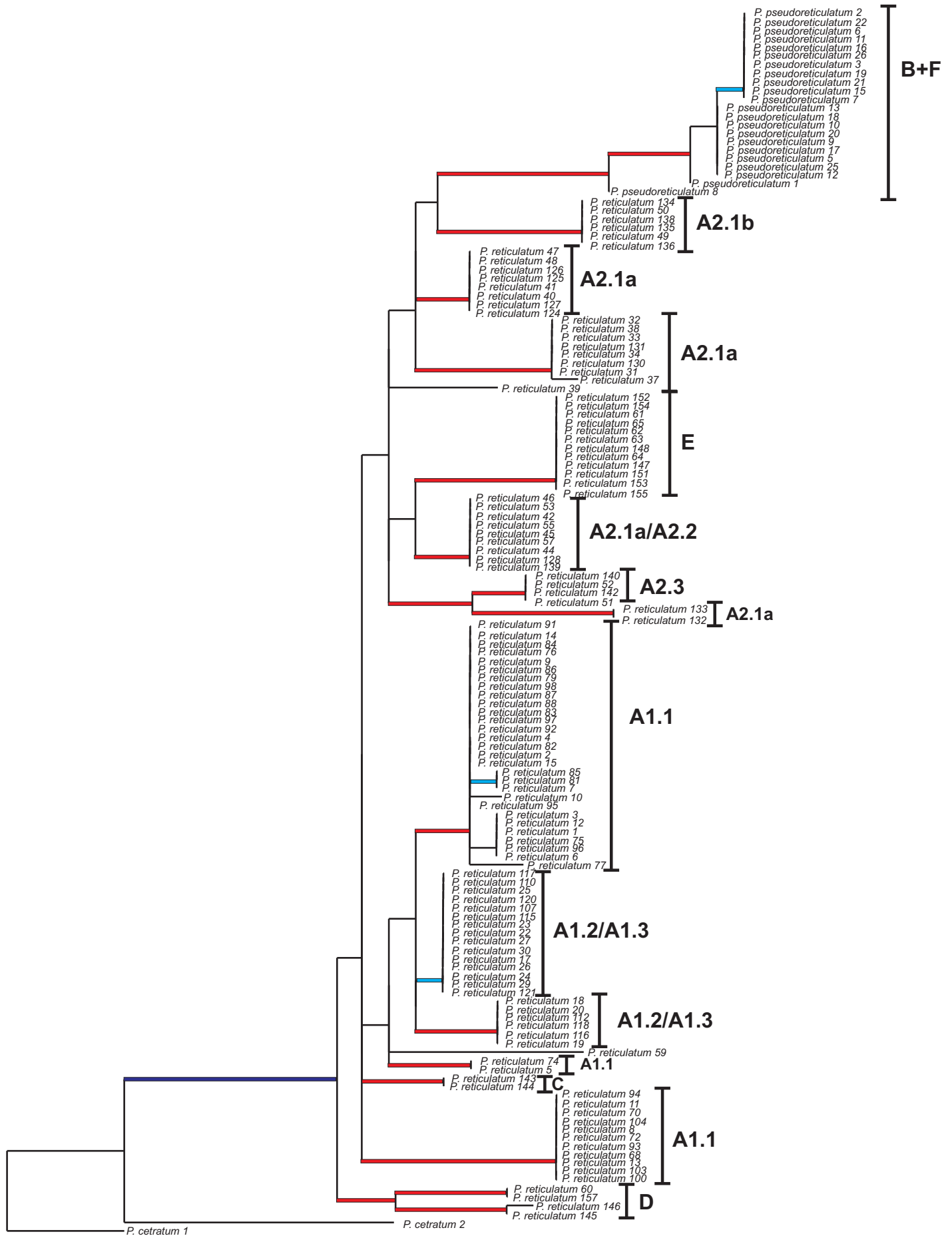

Supplement: S2 Fig — ML phylogenetic tree of Parmotrema reticulatum-Parmotrema pseudoreticulatum complex from MCM7 sequences. Branches that received strong support (bootstrap values ≥ 70%, posterior probabilities ≥ 0.95) in any of two analyses RaxML, and B/MCMC are in boldface. The branches that received strong support only in the ML bootstrap analysis are indicated by a blue boldface line, whereas branches that were strongly supported in both analyses are indicated by a red boldface line. (PDF) [file pone.0161031.s002.PDF]
