# Supplementary material for: Hidden Genetic Diversity in an Asexually Reproducing Lichen Forming Fungal Group
Source: PLoS One. 2016 Aug 11;11(8):e0161031. doi: 10.1371/journal.pone.0161031 (PMC4981466; doi:10.1371/journal.pone.0161031)
Supplement: S1 Table — Gen-Bank accession numbers for the two sampled loci: nuclear ribosomal internal transcribed spacer region (ITS), and DNA replication licensing factor MCM7 (mcm7). Newly generated sequences for this study are indicated in boldface. (DOC) [file pone.0161031.s003.doc]

| Species | Voucher Specimen | Locality | Collector (s) | GenBank accesion numbers nuITS | GenBank accesion numbers mcm7 |
| --- | --- | --- | --- | --- | --- |
| *P. reticulatum* 1 | MAF-Lich 6067 | Portugal, Sintra | *A. Crespo* & *X Jones* | AY586579 | **KX457802** |
| *P. reticulatum* 2 | MAF-Lich 16901 | Canary Islands, Gran Canaria | *A. Crespo, P. Cubas, A. Santos & P. K. Divakar* | JN166370 | **KX457803** |
| *P. reticulatum* 3 | MAF-Lich 16909 | Canary Islands, Hierro | *A. Crespo & S. Pérez-Ortega* | JN166371 | **KX457804** |
| *P. reticulatum* 4 | MAF-Lich 16900 | Canary Islands, Gran Canaria | *A. Crespo, P. Cubas, A. Santos & P. K. Divakar* | JN166372 | **KX457805** |
| *P. reticulatum* 5 | MAF-Lich 16898 | Fiji, Viti Levu Island | *H.T. Lumbsch* | JN166373 | **KX457806** |
| *P. reticulatum* 6 | MAF-Lich 16910 | Balearic Islands, Mallorca | *A. Crespo, P. K. Divakar, G. Amo, J. Núñez & S. Pina* | JN166375 | **KX457807** |
| *P. reticulatum* 7 | MAF-Lich 16143 | Morocco, Larache | *A. Crespo, P. K. Divakar, H. Tahiri, Dahmani, T. Lumbsch & G. Amo* | HM016953 | **KX457808** |
| *P. reticulatum* 8 | MAF-Lich 16918 | Canary Islands, La Gomera | *A. Crespo* | JN166376 | **KX457809** |
| *P. reticulatum* 9 | MAF-Lich 16904 | Canary Islands, Tenerife | *A. Crespo, P. Cubas, A. Santos & P. K. Divakar* | JN166374 | **KX457810** |
| *P. reticulatum* 10 | MAF-Lich 16917 | India, Tamil Nadu | *H. T. Lumbsch, D. K. Upreti, D. K. Divakar & J. Tandon* | JN166369 | **KX457811** |
| *P. reticulatum* 11 | MAF-Lich 16183 | Canary Islands, La Palma | *A. Crespo, R. Del Prado & A. Santos* | HM017064 | **KX457812** |
| *P. reticulatum* 12 | MAF-Lich 16175 | Canary Islands, La Palma | *A. Crespo, R. Del Prado & A. Santos* | HM017063 | **KX457813** |
| *P. reticulatum* 13 | MAF-Lich 16177 | Canary Islands, La Palma | *A. Crespo, R. Del Prado & A. Santos* | HM017062 | **KX457814** |
| *P. reticulatum* 14 | MAF-Lich 10265 | Canary Islands, Tenerife | *A. Crespo & P.K. Divakar* | AY642844 | **KX457815** |
| *P. reticulatum* 15 | MAF-Lich 10275 | Portugal, Evora | *A. Crespo & P.K. Divakar* | AY642836 | **KX457816** |
| *P. reticulatum* 16 | MAF-Lich 16893 | Republic of Peru, Canta | *A. Crespo, S. Pérez-Ortega & J. Núñez* | JN166377 | xxx |
| *P. reticulatum* 17 | MAF-Lich 16152 | Morocco, Rabat | *A. Crespo, P. K. Divakar, H. Tahiri, Dahmani & G. Amo* | HM016955 | **KX457817** |
| *P. reticulatum* 18 | MAF-Lich 16923 | Canary Islands, Gran Canaria | *A. Crespo* | JN166381 | **KX457818** |
| *P. reticulatum* 19 | MAF-Lich 10281 | Spain, Galicia | *P.K. Divakar* | HM016954 | **KX457819** |
| *P. reticulatum* 20 | MAF-Lich 16914 | Republic of Mozambique, Is Inhaca | *B. Roca-Valiente, E. Castoldi & A. Lumbreras* | JN166382 | **KX457820** |
| *P. reticulatum* 21 | MAF-Lich 7650 | Spain, Cádiz | *A. Crespo* | AY586578 | xxx |
| *P. reticulatum* 22 | MAF-Lich 16906 | Canary Islands, Gran Canaria | *A. Crespo, P. Cubas, A. Santos & P. K. Divakar* | JN166378 | **KX457821** |
| *P. reticulatum* 23 | MAF-Lich 10271 | Portugal, Portalegre | *A. Crespo & P.K. Divakar* | AY642838 | **KX457822** |
| *P. reticulatum* 24 | MAF-Lich 10270 | Portugal, Evora | *A. Crespo & P.K. Divakar* | AY642837 | **KX457823** |
| *P. reticulatum* 25 | K 301 | Kenya, Ngong | *V. Alstrup* | AY642845 | **KX457824** |
| *P. reticulatum* 26 | MAF-Lich 16915 | India, Tamil Nadu | *H. T. Lumbsch, D. K. Upreti, D. K. Divakar & J. Tandon* | JN166379 | **KX457825** |
| *P. reticulatum* 27 | MAF-Lich 10258 | China, Yunnan | *A. Crespo, O. Blanco & A. Argüello* | AY642818 | **KX457826** |
| *P. reticulatum* 28 | MAF-Lich 10259 | China, Yunnan | *A. Crespo, O. Blanco & A. Argüello* | AY642819 | xxx |
| *P. reticulatum* 29 | MAF-Lich 10257 | China, Yunnan | *A. Crespo, O. Blanco & A. Argüello* | AY642817 | **KX457827** |
| *P. reticulatum* 30 | MAF-Lich 16916 | India, Tamil Nadu | *H. T. Lumbsch, D. K. Upreti, D. K. Divakar & J. Tandon* | JN166380 | **KX457828** |
| *P. reticulatum* 31 | MAF-Lich 10164 | China, Yunnan | *A. Crespo, O. Blanco & A. Argüello* | AY586577 | **KX457829** |
| *P. reticulatum* 32 | MAF-Lich 16897 | Fiji, Viti Levu Island | *H. T. Lumbsch* | JN166383 | **KX457830** |
| *P. reticulatum* 33 | MAF-Lich 16895 | Japan, Tsukuba | *A. Crespo & P. K. Divakar* | JN166384 | **KX457831** |
| *P. reticulatum* 34 | MAF-Lich 16894 | Japan, Tsukuba | *A. Crespo, P. K. Divakar & Y. Ohmura* | JN166385 | **KX457832** |
| *P. reticulatum* 37 | MAF-Lich 10263 | China, Yunnan | *A. Crespo, O. Blanco & A. Argüello* | AY642824 | **KX457833** |
| *P. reticulatum* 38 | MAF-Lich 10262 | China, Yunnan | *A. Crespo, O. Blanco & A. Argüello* | AY642823 | **KX457834** |
| *P. reticulatum* 39 | MAF-Lich 10260 | China, Yunnan | *A. Crespo, O. Blanco & A. Argüello* | AY642821 | **KX457835** |
| *P. reticulatum* 40 | MAF-Lich 10285 | Spain, Pontevedra | *P.K. Divakar* | AY642832 | **KX457836** |
| *P. reticulatum* 41 | MAF-Lich 16911 | Balearic Islands, Mallorca | *A. Crespo, P. K. Divakar, G. Amo, J. Núñez & S. Pina* | JN166386 | **KX457837** |
| *P. reticulatum* 42 | MAF-Lich 16913 | Balearic Islands, Mallorca | *A. Crespo, P. K. Divakar, G. Amo, J. Núñez & S. Pina* | JN166387 | **KX457838** |
| *P. reticulatum* 43 | MAF-Lich 16899 | Canary Islands, Gran Canaria | *A. Crespo, P. Cubas, A. Santos & P. K. Divakar* | JN166388 | xxx |
| *P. reticulatum* 44 | MAF-Lich 16903 | Canary Islands, Tenerife | *A. Crespo, P. Cubas, A. Santos & P. K. Divakar* | JN166389 | **KX457839** |
| *P. reticulatum* 45 | MAF-Lich 16896 | Canary Islands, Tenerife | *A. Crespo, P. Cubas, A. Santos & P. K. Divakar* | JN166390 | **KX457840** |
| *P. reticulatum* 46 | MAF-Lich 16921 | Canary Islands, La Gomera | *A. Crespo* | JN166391 | **KX457841** |
| *P. reticulatum* 47 | MAF-Lich 16151 | Morocco, Larache | *A. Crespo, P. K. Divakar, H. Tahiri, Dahmani, T. Lumbsch & G. Amo* | HM017058 | **KX457842** |
| *P. reticulatum* 48 | MAF-Lich 16150 | Morocco, Larache | *A. Crespo, P. K. Divakar, H. Tahiri, Dahmani, T. Lumbsch & G. Amo* | HM017057 | **KX457843** |
| *P. reticulatum* 49 | MAF-Lich 10267 | Canary Islands, Tenerife | *A. Crespo* | AY642825 | **KX457844** |
| *P. reticulatum* 50 | MAF-Lich 16184 | Canary Islands, La Palma | *A. Crespo, R. Del Prado & A. Santos* | HM017059 | **KX457845** |
| *P. reticulatum* 51 | MAF-Lich 10264 | Canary Islands, Tenerife | *A. Crespo* | HM017060 | **KX457846** |
| *P. reticulatum* 52 | MAF-Lich 16902 | Canary Islands, Tenerife | *A. Crespo, P. Cubas, A. Santo & P. K. Divakar* | JN166397 | **KX457847** |
| *P. reticulatum* 53 | MAF-Lich 16920 | Canary Islands, La Gomera | *A. Crespo* | JN166395 | **KX457848** |
| *P. reticulatum* 54 | MAF-Lich 16926 | France, Bretagne | *P. Cubas & R. Oyarzum* | JN166393 | xxx |
| *P. reticulatum* 55 | MAF-Lich 16919 | Canary Islands, La Gomera | *A. Crespo* | JN166394 | **KX457849** |
| *P. reticulatum* 56 | MAF-Lich 16925 | Canary Islands, La Gomera | *A. Crespo* | JN166396 | xxx |
| *P. reticulatum* 57 | MAF-Lich 16924 | Canary Islands, La Gomera | *A. Crespo* | JN166392 | **KX457850** |
| *P. reticulatum* 59 | MAF-Lich 16891 | Chile, Tierra de Fuego | *S. Pérez-Ortega* | JN166401 | **KX457851** |
| *P. reticulatum* 60 | MAF-Lich 16892 | Chile, Tierra de Fuego | *S. Pérez-Ortega* | JN166402 | **KX457852** |
| *P. reticulatum* 61 | MAF-Lich 16182 | Canary Islands, La Palma | *A. Crespo, R. Del Prado & A. Santos* | HM016957 | **KX457853** |
| *P. reticulatum* 62 | MAF-Lich 16922 | Canary Islands, Gran Canaria | *A. Crespo* | JN166398 | **KX457854** |
| *P. reticulatum* 63 | MAF-Lich 16180 | Canary Islands, La Palma | *A. Crespo, R. Del Prado & A. Santos* | HM016960 | **KX457855** |
| *P. reticulatum* 64 | MAF-Lich 16178 | Canary Islands, La Palma | *A. Crespo, R. Del Prado & A. Santos* | HM016959 | **KX457856** |
| *P. reticulatum* 65 | MAF-Lich 16181 | Canary Islands, La Palma | *A. Crespo, R. Del Prado & A. Santos* | HM016958 | **KX457857** |
| *P. reticulatum* 68 | MAF-Lich 20567 | Portugal, Madeira | *P.K. Divakar & M. Talavera* | **KX457708** | **KX457858** |
| *P. reticulatum* 69 | MAF-Lich 17002 | Portugal, Sintra | *P.K. Divakar, C. Ruibal & A. Agudo* | **KX457709** | xxx |
| *P. reticulatum* 70 | MAF-Lich16957 | Canary Islands, Hierro | *A. Crespo & S. Pérez Ortega* | **KX457710** | **KX457859** |
| *P. reticulatum* 71 | MAF-Lich 16994 | Canary Islands, Gran Canaria | *A. Crespo, P. Cubas, A. Santos & P.K. Divakar* | **KX457711** | xxx |
| *P. reticulatum* 72 | MAF-Lich 16960 | Canary Islands, La Gomera | *A. Crespo* | **KX457712** | **KX457860** |
| *P. reticulatum* 73 | MAF-Lich 17001 | Portugal, Sintra | *P.K. Divakar, C. Ruibal & A. Agudo* | **KX457713** | xxx |
| *P. reticulatum* 74 | MAF-Lich 20588 | Australia, Victoria | *A. Crespo, R. Gavilán & P. K. Divakar* | **KX457714** | **KX457861** |
| *P. reticulatum* 75 | MAF-Lich 20581 | Portugal, Azores | *A. Crespo, M.A. Carrasco & P.K. Divakar* | **KX457715** | **KX457862** |
| *P. reticulatum* 76 | MAF-Lich 17000 | Portugal, Sintra | *P.K. Divakar, C. Ruibal & A. Agudo* | **KX457716** | **KX457863** |
| *P. reticulatum* 77 | MAF-Lich 10291 | Spain, Málaga | *A. Crespo* | AY642820 | **KX457864** |
| *P. reticulatum* 78 | MAF-Lich 16989 | Canary Islands, Gran Canaria | *A. Crespo, P. Cubas, A. Santos & P.K. Divakar* | **KX457717** | xxx |
| *P. reticulatum* 79 | MAF-Lich 10272 | Portugal, Santarem | *A. Crespo* | AY642827 | **KX457865** |
| *P. reticulatum* 80 | MAF-Lich 16993 | Canary Islands, Gran Canaria | *A. Crespo, P. Cubas, A. Santos & P.K. Divakar* | **KX457718** | xxx |
| *P. reticulatum* 81 | MAF-Lich 16999 | Portugal, Sintra | *P.K. Divakar, C. Ruibal & A. Agudo* | **KX457719** | **KX457866** |
| *P. reticulatum* 82 | MAF-Lich 16995 | Portugal, Sintra | *P.K. Divakar, C. Ruibal & A. Agudo* | **KX457720** | **KX457867** |
| *P. reticulatum* 83 | MAF-Lich 16988 | Canary Islands, Gran Canaria | *A. Crespo, P. Cubas, A. Santos & P.K. Divakar* | **KX457721** | **KX457868** |
| *P. reticulatum* 84 | MAF-Lich 20584 | Australia, Victoria | *A. Crespo, R. Gavilán & P. K. Divakar* | **KX457722** | **KX457869** |
| *P. reticulatum* 85 | MAF-Lich 16998 | Portugal, Sintra | *P.K. Divakar, C. Ruibal & A. Agudo* | **KX457723** | **KX457870** |
| *P. reticulatum* 86 | MAF-Lich 20570 | Portugal, Madeira | *P.K. Divakar & M. Talavera* | **KX457724** | **KX457871** |
| *P. reticulatum* 87 | MAF-Lich 16979 | Canary Islands, Tenerife | *A. Crespo, P. Cubas, A. Santos & P.K. Divakar* | **KX457725** | **KX457872** |
| *P. reticulatum* 88 | MAF-Lich 20579 | Portugal, Azores | *A. Crespo, M.A. Carrasco & P.K. Divakar* | **KX457726** | **KX457873** |
| *P. reticulatum* 89 | MAF-Lich 16962 | Canary Islands, La Gomera | *A. Crespo* | **KX457727** | xxx |
| *P. reticulatum* 90 | MAF-Lich 16973 | Canary Islands, Tenerife | *A. Crespo, P. Cubas, A. Santos & P.K. Divakar* | **KX457728** | xxx |
| *P. reticulatum* 91 | MAF-Lich 10284 | Spain, Cies Islands | *P.K. Divakar* | AY642831 | **KX457874** |
| *P. reticulatum* 92 | MAF-Lich 16992 | Canary Islands, Gran Canaria | *A. Crespo, P. Cubas, A. Santos & P.K. Divakar* | **KX457729** | **KX457875** |
| *P. reticulatum* 93 | MAF-Lich 20577 | Portugal, Azores | *A. Crespo, M.A. Carrasco & P.K. Divakar* | **KX457730** | **KX457876** |
| *P. reticulatum* 94 | MAF-Lich 20568 | Portugal, Madeira | *P.K. Divakar & M. Talavera* | **KX457731** | **KX457877** |
| *P. reticulatum* 95 | MAF-Lich 20572 | Portugal, Madeira | *P.K. Divakar & M. Talavera* | **KX457732** | **KX457878** |
| *P. reticulatum* 96 | MAF-Lich 20554 | Portugal, Algarve | *P.K. Divakar, C. Ruibal & A. Agudo* | **KX457733** | **KX457879** |
| *P. reticulatum* 97 | MAF-Lich 16990 | Canary Islands, Gran Canaria | *A. Crespo, P. Cubas, A. Santos & P.K. Divakar* | **KX457734** | **KX457880** |
| *P. reticulatum* 98 | MAF-Lich 16991 | Canary Islands, Gran Canaria | *A. Crespo, P. Cubas, A. Santos & P.K. Divakar* | **KX457735** | **KX457881** |
| *P. reticulatum* 99 | MAF-Lich 16956 | Canary Islands, Hierro | *A. Crespo & S. Pérez Ortega* | **KX457736** | xxx |
| *P. reticulatum* 100 | MAF-Lich 16961 | Canary Islands, La Gomera | *A. Crespo* | **KX457737** | **KX457882** |
| *P. reticulatum* 101 | MAF-Lich 16965 | Canary Islands, La Palma | *A. Crespo, P. Cubas, A. Santos & P.K. Divakar* | **KX457738** | xxx |
| *P. reticulatum* 102 | MAF-Lich 16958 | Canary Islands, La Gomera | *A. Crespo* | **KX457739** | xxx |
| *P. reticulatum* 103 | MAF-Lich 20573 | Portugal, Azores | *A. Crespo, M.A. Carrasco & P.K. Divakar* | **KX457740** | **KX457883** |
| *P. reticulatum* 104 | MAF-Lich 20578 | Portugal, Azores | *A. Crespo, M.A. Carrasco & P.K. Divakar* | **KX457741** | **KX457884** |
| *P. reticulatum* 105 | MAF-Lich 10273 | Portugal, Santarem | *A. Crespo* | AY642826 | xxx |
| *P. reticulatum* 106 | MAF-Lich 20590 | Peru, Amazonas | *J. Nuñez Zapata* | **KX457742** | xxx |
| *P. reticulatum* 107 | MAF-Lich 20555 | Portugal, Algarve | *P.K. Divakar, C. Ruibal & A. Agudo* | **KX457743** | **KX457885** |
| *P. reticulatum* 108 | MAF-Lich 16997 | Portugal, Sintra | *P.K. Divakar, C. Ruibal & A. Agudo* | **KX457744** | xxx |
| *P. reticulatum* 109 | MAF-Lich 20561 | Morocco, Rabat | *A. Crespo, P. K. Divakar, H. Tahiri, Dahmani & G. Amo* | **KX457745** | xxx |
| *P. reticulatum* 110 | MAF-Lich 17004 | Portugal, Algarve | *P.K. Divakar, C. Ruibal & A. Agudo* | **KX457746** | **KX457886** |
| *P. reticulatum* 111 | MAF-Lich 16972 | Canary Islands, Tenerife | *A. Crespo, P. Cubas, A. Santos & P.K. Divakar* | **KX457747** | xxx |
| *P. reticulatum* 112 | MAF-Lich 16959 | Canary Islands, La Gomera | *A. Crespo* | **KX457748** | **KX457887** |
| *P. reticulatum* 113 | MAF-Lich 20585 | Australia, Victoria | *A. Crespo, R. Gavilán & P. K. Divakar* | **KX457749** | xxx |
| *P. reticulatum* 114 | MAF-Lich 20587 | Australia, Victoria | *A. Crespo, R. Gavilán & P. K. Divakar* | **KX457750** | xxx |
| *P. reticulatum* 115 | MAF-Lich 20563 | Canary Islands, Tenerife | *A. Crespo, P. Cubas, A. Santos & P.K. Divakar* | **KX457751** | **KX457888** |
| *P. reticulatum* 116 | MAF-Lich 16970 | Canary Islands, La Palma | *A. Crespo, P. Cubas, A. Santos & P.K. Divakar* | **KX457752** | **KX457889** |
| *P. reticulatum* 117 | MAF-Lich 16955 | Morocco, Rabat | *H. Tahiri* | **KX457753** | **KX457890** |
| *P. reticulatum* 118 | MAF-Lich 20556 | Portugal, Algarve | *P.K. Divakar, C. Ruibal & A. Agudo* | **KX457754** | **KX457891** |
| *P. reticulatum* 119 | MAF-Lich 20562 | Morocco, Rabat | *A. Crespo, P. K. Divakar, H. Tahiri, Dahmani & G. Amo* | **KX457755** | xxx |
| *P. reticulatum* 120 | MAF-Lich 16975 | Canary Islands, Tenerife | *A. Crespo, P. Cubas, A. Santos & P.K. Divakar* | **KX457756** | **KX457892** |
| *P. reticulatum* 121 | MAF-Lich 20558 | Portugal, Algarve | *P.K. Divakar, C. Ruibal & A. Agudo* | **KX457757** | **KX457893** |
| *P. reticulatum* 122 | MAF-Lich 16969 | Canary Islands, La Palma | *A. Crespo, P. Cubas, A. Santos & P.K. Divakar* | **KX457758** | xxx |
| *P. reticulatum* 123 | MAF-Lich 16968 | Canary Islands, La Palma | *A. Crespo, P. Cubas, A. Santos & P.K. Divakar* | **KX457759** | xxx |
| *P. reticulatum* 124 | MAF-Lich 10283 | Spain, Galicia | *P.K. Divakar* | AY642846 | **KX457894** |
| *P. reticulatum* 125 | MAF-Lich 16996 | Portugal, Sintra | *P.K. Divakar, C. Ruibal & A. Agudo* | **KX457760** | **KX457895** |
| *P. reticulatum* 126 | MAF-Lich 10282 | Spain, Pontevedra | *P.K. Divakar* | AY642833 | **KX457896** |
| *P. reticulatum* 127 | MAF-Lich 10280 | Portugal, Evora | *A. Crespo & P.K. Divakar* | AY642835 | **KX457897** |
| *P. reticulatum* 128 | MAF-Lich 20566 | Canary Islands, El Hierro | *A. Crespo, P. Cubas, A. Santos & P.K. Divakar* | **KX457761** | **KX457898** |
| *P. reticulatum* 129 | MAF-Lich 17003 | Portugal, Algarve | *P.K. Divakar, C. Ruibal & A. Agudo* | **KX457762** | xxx |
| *P. reticulatum* 130 | MAF-Lich 20589 | Japan, Tsukuba | *A. Crespo &P. K. Divakar* | **KX457763** | **KX457899** |
| *P. reticulatum* 131 | MAF-Lich 10261 | China, Yunnan | *A. Crespo, O. Blanco & A. Argüello* | AY642822 | **KX457900** |
| *P. reticulatum* 132 | MAF-Lich 16978 | Canary Islands, Tenerife | *A. Crespo, P. Cubas, A. Santos & P.K. Divakar* | **KX457764** | **KX457901** |
| *P. reticulatum* 133 | MAF-Lich 20564 | Canary Islands, Tenerife | *A. Crespo, P. Cubas, A. Santos & P.K. Divakar* | **KX457765** | **KX457902** |
| *P. reticulatum* 134 | MAF-Lich 20574 | Portugal, Azores | *A. Crespo, M.A. Carrasco & P.K. Divakar* | **KX457766** | **KX457903** |
| *P. reticulatum* 135 | MAF-Lich 16974 | Canary Islands, Tenerife | *A. Crespo, P. Cubas, A. Santos & P.K. Divakar* | **KX457767** | **KX457904** |
| *P. reticulatum* 136 | MAF-Lich 20580 | Portugal, Azores | *A. Crespo, M.A. Carrasco & P.K. Divakar* | **KX457768** | **KX457905** |
| *P. reticulatum* 137 | MAF-Lich 20582 | Portugal, Azores | *A. Crespo, M.A. Carrasco & P.K. Divakar* | **KX457769** | xxx |
| *P. reticulatum* 138 | MAF-Lich 10268 | Canary Islands, Tenerife | *A. Crespo* | AY642834 | **KX457906** |
| *P. reticulatum* 139 | MAF-Lich 16963 | Canary Islands, La Gomera | *A. Crespo* | **KX457770** | **KX457907** |
| *P. reticulatum* 140 | MAF-Lich 10266 | Canary Islands, Tenerife | *A. Crespo* | HM017061 | **KX457908** |
| *P. reticulatum* 141 | MAF-Lich 20571 | Portugal, Madeira | *P.K. Divakar & M. Talavera* | **KX457771** | xxx |
| *P. reticulatum* 142 | MAF-Lich 20569 | Portugal, Madeira | *P.K. Divakar & M. Talavera* | **KX457772** | **KX457909** |
| *P. reticulatum* 143 | MAF-Lich 16987 | Canary Islands, Gran Canaria | *A. Crespo, P. Cubas, A. Santos & P.K. Divakar* | **KX457773** | **KX457910** |
| *P. reticulatum* 144 | MAF-Lich 16905 | Canary Islands, Gran Canaria | *A. Crespo, P. Cubas, A. Santo & P. K. Divakar* | JN166400 | **KX457911** |
| *P. reticulatum* 145 | MAF-Lich 20583 | Australia, Victoria | *A. Crespo, R. Gavilán & P. K. Divakar* | **KX457774** | **KX457912** |
| *P. reticulatum* 146 | MAF-Lich 20586 | Australia, Victoria | *A. Crespo, R. Gavilán & P. K. Divakar* | **KX457775** | **KX457913** |
| *P. reticulatum* 147 | MAF-Lich 16985 | Canary Islands, Gran Canaria | *A. Crespo, P. Cubas, A. Santo & P. K. Divakar* | **KX457776** | **KX457914** |
| *P. reticulatum* 148 | MAF-Lich 16976 | Canary Islands, Tenerife | *A. Crespo, P. Cubas, A. Santo & P. K. Divakar* | **KX457777** | **KX457915** |
| *P. reticulatum* 149 | MAF-Lich 16982 | Canary Islands, Gran Canaria | *A. Crespo, P. Cubas, A. Santo & P. K. Divakar* | **KX457778** | xxx |
| *P. reticulatum* 150 | MAF-Lich 16983 | Canary Islands, Gran Canaria | *A. Crespo, P. Cubas, A. Santo & P. K. Divakar* | **KX457779** | xxx |
| *P. reticulatum* 151 | MAF-Lich 16964 | Canary Islands, La Palma | *A. Crespo, P. Cubas, A. Santo & P. K. Divakar* | **KX457780** | **KX457916** |
| *P. reticulatum* 152 | MAF-Lich 16986 | Canary Islands, Gran Canaria | *A. Crespo, P. Cubas, A. Santo & P. K. Divakar* | **KX457781** | **KX457917** |
| *P. reticulatum* 153 | MAF-Lich 16977 | Canary Islands, Gran Canaria | *A. Crespo, P. Cubas, A. Santo & P. K. Divakar* | **KX457782** | **KX457918** |
| *P. reticulatum* 154 | MAF-Lich 16984 | Canary Islands, Gran Canaria | *A. Crespo, P. Cubas, A. Santo & P. K. Divakar* | **KX457783** | **KX457919** |
| *P. reticulatum* 155 | MAF-Lich 16981 | Canary Islands, Gran Canaria | *A. Crespo, P. Cubas, A. Santo & P. K. Divakar* | **KX457784** | **KX457920** |
| *P. reticulatum* 156 | MAF-Lich 16980 | Canary Islands, Gran Canaria | *A. Crespo, P. Cubas, A. Santo & P. K. Divakar* | **KX457785** | xxx |
| *P. reticulatum* 157 | MAF-Lich 10290 | Chile, Los Rios | *L. G. Sancho* | HM016961 | **KX457921** |
| *P. pseudoreticulatum* 1 | MAF-Lich 10287 | South Africa, Western Cape | *A. Aparicio, A. R. Burgaz, M. A. Carrasco & X. Giráldez* | AY642828 | **KX457922** |
| *P. pseudoreticulatum 2* | MAF-Lich 16144 | Morocco, Chaouen | *A. Crespo, P. K. Divakar, H. Tahiri, Dahmani, T. Lumbsch & G. Amo* | HM017055 | **KX457923** |
| *P. pseudoreticulatum* 3 | MAF-Lich 16149 | Morocco, Rabat | *A. Crespo, P. K. Divakar, H. Tahiri, Dahmani & G. Amo* | HM017056 | **KX457924** |
| *P. pseudoreticulatum* 4 | MAF-Lich 7650A | Spain, Cádiz | *A. Crespo* | HM017054 | xxx |
| *P. pseudoreticulatum* 5 | MAF-Lich 16185 | Canary Islands, La Palma | *A. Crespo, R. Del Prado & A. Santos* | HM017053 | **KX457925** |
| *P. pseudoreticulatum* 6 | MAF-Lich 10277 | Portugal, Estremadura | *C. García* | AY642842 | **KX457926** |
| *P. pseudoreticulatum* 7 | MAF-Lich 10278 | Portugal, Estremadura | *C. García* | AY642841 | **KX457927** |
| *P. pseudoreticulatum* 8 | MAF-Lich 10289 | South Africa, Western Cape | *A. Aparicio, A. R. Burgaz, M. A. Carrasco & X. Giráldez* | AY642830 | **KX457928** |
| *P. pseudoreticulatum* 9 | MAF-Lich 10288 | South Africa, Western Cape | *A. Aparicio, A. R. Burgaz, M. A. Carrasco & X. Giráldez* | AY642829 | **KX457929** |
| *P pseudoreticulatum* 10 | MAF-Lich 10276 | Portugal, Estremadura | *C. García* | AY642839 | **KX457930** |
| *P. pseudoreticulatum* 11 | MAF-Lich 16912 | Balearic Islands, Mallorca | *A. Crespo, P. K. Divakar, G. Amo, J. Núñez & S. Pina* | JN166399 | **KX457931** |
| *P pseudoreticulatum* 12 | MAF-Lich 10292 | Portugal, Estremadura | *C. García* | AY642840 | **KX457932** |
| *P. pseudoreticulatum* 13 | MAF-Lich 20576 | Portugal, Azores | *A. Crespo, M.A. Carrasco & P.K. Divakar* | **KX457786** | **KX457933** |
| *P. pseudoreticulatum* 14 | MAF-Lich 17010 | Portugal, Algarve | *P.K. Divakar, C. Ruibal & A. Agudo* | **KX457787** | xxx |
| *P. pseudoreticulatum* 15 | MAF-Lich 17007 | Portugal, Algarve | *P.K. Divakar, C. Ruibal & A. Agudo* | **KX457788** | **KX457934** |
| *P. pseudoreticulatum* 16 | MAF-Lich 17009 | Portugal, Algarve | *P.K. Divakar, C. Ruibal & A. Agudo* | **KX457789** | **KX457935** |
| *P. pseudoreticulatum* 17 | MAF-Lich 16967 | Canary Islands, La Palma | *A. Crespo, P. Cubas, A. Santos & P.K. Divakar* | **KX457790** | **KX457936** |
| *P. pseudoreticulatum* 18 | MAF-Lich 17008 | Portugal, Algarve | *P.K. Divakar, C. Ruibal & A. Agudo* | **KX457791** | **KX457937** |
| *P. pseudoreticulatum* 19 | MAF-Lich 16966 | Canary Islands, La Palma | *A. Crespo, P. Cubas, A. Santos & P.K. Divakar* | **KX457792** | **KX457938** |
| *P. pseudoreticulatum* 20 | MAF-Lich 20559 | Portugal, Algarve | *P.K. Divakar, C. Ruibal & A. Agudo* | **KX457793** | **KX457939** |
| *P. pseudoreticulatum* 21 | MAF-Lich 20575 | Portugal, Azores | *A. Crespo, M.A. Carrasco & P.K. Divakar* | **KX457794** | **KX457940** |
| *P. pseudoreticulatum* 22 | MAF-Lich 17006 | Portugal, Algarve | *P.K. Divakar, C. Ruibal & A. Agudo* | **KX457795** | **KX457941** |
| *P. pseudoreticulatum* 23 | MAF-Lich 20560 | Morocco, Rabat | *A. Crespo, P. K. Divakar, H. Tahiri, Dahmani & G. Amo* | **KX457796** | xxx |
| *P. pseudoreticulatum* 24 | MAF-Lich 20557 | Portugal, Algarve | *P.K. Divakar, C. Ruibal & A. Agudo* | **KX457797** | xxx |
| *P. pseudoreticulatum 25* | MAF-Lich 17005 | Portugal, Algarve | *P.K. Divakar, C. Ruibal & A. Agudo* | **KX457798** | **KX457942** |
| *P. pseudoreticulatum* 26 | MAF-Lich 16971 | Canary Islands, La Palma | *A. Crespo, P. Cubas, A. Santos & P.K. Divakar* | **KX457799** | **KX457943** |
| *P. pseudoreticulatum* 27 | MAF-Lich 20565 | Canary Islands, La Palma | *A. Crespo, P. Cubas, A. Santos & P.K. Divakar* | **KX457800** | xxx |
| *P. pseudoreticulatum* 28 | MAF-Lich 16954 | Morocco, Rabat | *H. Tahiri* | **KX457801** | xxx |
| *P. aff. cetratum* 1 | Lucking 15593 A | Costa Rica,  Puntarenas | R. Lucking | AY642848 | **KX457944** |
| *P. aff. cetratum 2* | Lucking 15116 A | Costa Rica,  Puntarenas | R. Lucking | AY642850 | **KX457945** |
