# Supplementary material for: Hidden Genetic Diversity in an Asexually Reproducing Lichen Forming Fungal Group
Source: PLoS One. 2016 Aug 11;11(8):e0161031. doi: 10.1371/journal.pone.0161031 (PMC4981466; doi:10.1371/journal.pone.0161031)
Supplement: S2 Table — Clades do not recovered in the topology are indicated with an asterisk (*). (DOC) [file pone.0161031.s004.doc]

| **Clades** | Statistical support on nodes recovered | | |
| --- | --- | --- | --- |
|  | **Concatenated topology** | **ITS topology** | **mcm7 topology** |
| **A1** | ML / MrBayes | Not supported | * |
| A1.1 | ML / MrBayes | ML / MrBayes | Splits into 3 supported monophyletic clades |
| A.1.2 | Not supported | ML /MrBayes | Samples from both clades are intermixed in two supported clades |
| A1.3 | MrBayes | MrBayes |
| **A2** | ML / MrBayes | ML / MrBayes | * |
| A2.1 | Not supported | Mr Bayes | Split into 5 supported monophyletic clades |
| A2.1a | Not supported | ML / MrBayes | Split into 4 monophyletic groups |
| A2.1b | ML / MrBayes | ML / MrBayes | ML / MrBayes |
| A2.2 | ML / MrBayes | ML / MrBayes | Intermixed with samples of clade A2.1a |
| A2.3 | ML / MrBayes | ML / MrBayes | ML / MrBayes |
| **B** | ML / MrBayes | ML / MrBayes | B+F ML / MrBayes |
| **C** | ML / MrBayes | ML / MrBayes | ML / MrBayes |
| **D** | ML / MrBayes | ML / MrBayes | ML / MrBayes |
| **E** | ML / MrBayes | ML / MrBayes | ML / MrBayes |
| **F** | ML / MrBayes | ML / MrBayes | B+F ML / MrBayes |
