# Supplementary material for: Hidden Genetic Diversity in an Asexually Reproducing Lichen Forming Fungal Group
Source: PLoS One. 2016 Aug 11;11(8):e0161031. doi: 10.1371/journal.pone.0161031 (PMC4981466; doi:10.1371/journal.pone.0161031)
Supplement: S1 Fig — ML phylogenetic tree of Parmotrema reticulatum-Parmotrema pseudoreticulatum complex from ITS sequences. Branches that received strong support (bootstrap values ≥ 70%, posterior probabilities ≥ 0.95) in any of two analyses RaxML, and B/MCMC are in boldface. The branch that received strong support only in the ML bootstrap analysis is indicated by a blue boldface line, whereas branches that received strong support only in the B/MCMC analysis are indicated by a black boldface line. Branches that were strongly supported in both analyses are indicated by a red boldface line. (PDF) [file pone.0161031.s001.PDF]

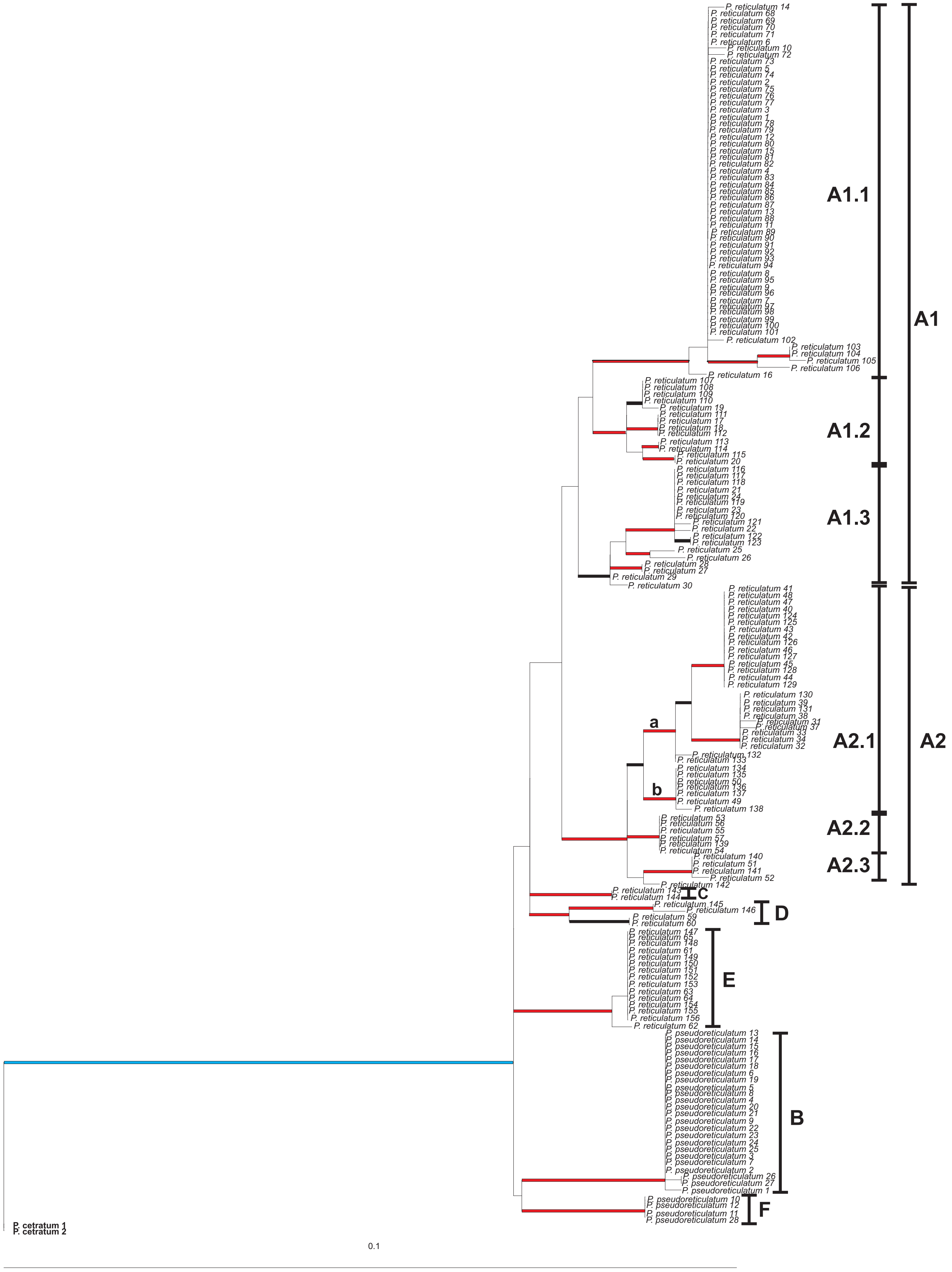
P. reticulatum 14
P. reticulatum 68
P. reticulatum 69
P. reticulatum 70
P. reticulatum 71
P. reticulatum 6
P. reticulatum 10
P. reticulatum 72
P. reticulatum 73
P. reticulatum 5
P. reticulatum 74
P. reticulatum 2
P. reticulatum 75
P. reticulatum 76
P. reticulatum 77
P. reticulatum 3
P. reticulatum 1
P. reticulatum 78
P. reticulatum 79
P. reticulatum 12
P. reticulatum 80
P. reticulatum 15
P. reticulatum 81
P. reticulatum 82
P. reticulatum 4
P. reticulatum 83
P. reticulatum 84
P. reticulatum 85
P. reticulatum 86
P. reticulatum 87
P. reticulatum 13
P. reticulatum 88
P. reticulatum 11
P. reticulatum 89
P. reticulatum 90
P. reticulatum 91
P. reticulatum 92
P. reticulatum 93
P. reticulatum 94
P. reticulatum 8
P. reticulatum 95
P. reticulatum 9
P. reticulatum 96
P. reticulatum 7
P. reticulatum 97
P. reticulatum 98
P. reticulatum 99
P. reticulatum 100
P. reticulatum 101
P. reticulatum 102
P. reticulatum 103
P. reticulatum 104
P. reticulatum 105
P. reticulatum 106
P. reticulatum 16
P. reticulatum 107
P. reticulatum 108
P. reticulatum 109
P. reticulatum 110
P. reticulatum 19
P. reticulatum 111
P. reticulatum 17
P. reticulatum 18
P. reticulatum 112
P. reticulatum 113
P. reticulatum 114
P. reticulatum 115
P. reticulatum 20
P. reticulatum 116
P. reticulatum 117
P. reticulatum 118
P. reticulatum 21
P. reticulatum 24
P. reticulatum 119
P. reticulatum 23
P. reticulatum 120
P. reticulatum 121
P. reticulatum 22
P. reticulatum 122
P. reticulatum 123
P. reticulatum 25
P. reticulatum 26
P. reticulatum 28
P. reticulatum 27
P. reticulatum 29
P. reticulatum 30
P. reticulatum 41
P. reticulatum 48
P. reticulatum 47
P. reticulatum 40
P. reticulatum 124
P. reticulatum 125
P. reticulatum 43
P. reticulatum 42
P. reticulatum 126
P. reticulatum 46
P. reticulatum 127
P. reticulatum 45
P. reticulatum 128
P. reticulatum 44
P. reticulatum 129
P. reticulatum 130
P. reticulatum 39
P. reticulatum 131
P. reticulatum 38
P. reticulatum 31
P. reticulatum 37
P. reticulatum 33
P. reticulatum 34
P. reticulatum 32
P. reticulatum 132
P. reticulatum 133
P. reticulatum 134
P. reticulatum 135
P. reticulatum 50
P. reticulatum 136
P. reticulatum 137
P. reticulatum 49
P. reticulatum 138
P. reticulatum 53
P. reticulatum 56
P. reticulatum 55
P. reticulatum 57
P. reticulatum 139
P. reticulatum 54
P. reticulatum 140
P. reticulatum 51
P. reticulatum 141
P. reticulatum 52
P. reticulatum 142
P. reticulatum 143
P. reticulatum 144
P. reticulatum 145
P. reticulatum 146
P. reticulatum 59
P. reticulatum 60
P. reticulatum 147
P. reticulatum 65
P. reticulatum 148
P. reticulatum 61
P. reticulatum 149
P. reticulatum 150
P. reticulatum 151
P. reticulatum 152
P. reticulatum 153
P. reticulatum 63
P. reticulatum 64
P. reticulatum 154
P. reticulatum 155
P. reticulatum 156
P. reticulatum 62
P. pseudoreticulatum 13
P. pseudoreticulatum 14
P. pseudoreticulatum 15
P. pseudoreticulatum 16
P. pseudoreticulatum 17
P. pseudoreticulatum 18
P. pseudoreticulatum 6
P. pseudoreticulatum 19
P. pseudoreticulatum 5
P. pseudoreticulatum 8
P. pseudoreticulatum 4
P. pseudoreticulatum 20
P. pseudoreticulatum 21
P. pseudoreticulatum 9
P. pseudoreticulatum 22
P. pseudoreticulatum 23
P. pseudoreticulatum 24
P. pseudoreticulatum 25
P. pseudoreticulatum 3
P. pseudoreticulatum 7
P. pseudoreticulatum 2
P. pseudoreticulatum 26
P. pseudoreticulatum 27
P. pseudoreticulatum 1
P. pseudoreticulatum 10
P. pseudoreticulatum 12
P. pseudoreticulatum 11
P. pseudoreticulatum 28
P. cetratum 1
P. cetratum 2
A1.1
A1.2
A1.3
A1
a
b
A2.1
A2.2
A2.3
A2
C
D
E
B
F
0.1
